# Beneficial Effects of Vitamin K Status on Glycemic Regulation and Diabetes Mellitus: A Mini-Review

**DOI:** 10.3390/nu12082485

**Published:** 2020-08-18

**Authors:** Hsin-Jung Ho, Michio Komai, Hitoshi Shirakawa

**Affiliations:** 1 Laboratory of Nutrition, Graduate School of Agricultural Science, Tohoku University, Sendai 980-8572, Japan; mkomai@m.tohoku.ac.jp (M.K.); shirakah@tohoku.ac.jp (H.S.); 2 International Education and Research Center for Food Agricultural Immunology, Graduate School of Agricultural Science, Tohoku University, Sendai 980-8572, Japan

**Keywords:** vitamin K, insulin sensitivity, glycemic status, diabetes mellitus

## Abstract

Type 2 diabetes mellitus is a chronic disease that is characterized by hyperglycemia, insulin resistance, and dysfunctional insulin secretion. Glycemic control remains a crucial contributor to the progression of type 2 diabetes mellitus as well as the prevention or delay in the onset of diabetes-related complications. Vitamin K is a fat-soluble vitamin that plays an important role in the regulation of the glycemic status. Supplementation of vitamin K may reduce the risk of diabetes mellitus and improve insulin sensitivity. This mini-review summarizes the recent insights into the beneficial effects of vitamin K and its possible mechanism of action on insulin sensitivity and glycemic status, thereby suppressing the progression of diabetes mellitus.

## 1. Introduction

Type 2 diabetes mellitus (T2DM) is a chronic health condition that occurs when insulin secretion is impaired and manifests through features associated with insulin resistance. Several pathophysiologic defects, including the disequilibrium of insulin and glucagon secretory capacities of pancreatic α- and β-cells, hepatic steatosis, insulin resistance, reduced incretin secretion in the small intestine, and impaired glucose uptake in the peripheral tissues, cause the progressive hyperglycemia of T2DM [1,2]. Glycemic control remains a crucial contributor in the progression of T2DM and the prevention or delay in the onset of diabetes-related microvascular and macrovascular complications. Glucose-dependent insulinotropic peptide (GIP) and glucagon-like peptide-1 (GLP-1) are incretins released from gut enteroendocrine cells, which play a major role in the postprandial regulation of insulin secretion through augmentation of insulin, suppression of glucagon secretion, and decrease of endogenous glucose production [3,4,5,6,7]. Studies have highlighted the possibility of the incretin-based treatment strategies [8,9]. Recently, GLP-1 receptor agonists and sodium-glucose cotransporter-2 (SGLT2) inhibitors are considered as the glucose-lowering drugs that have moderated benefits in reducing cardiovascular risk among T2DM patients [10]. In addition, a review integrated the genome-wide association studies (GWAS) of T2DM, implicating around 250 associated genetic variations to T2DM. GWAS is a helpful approach to understand the interaction between β-cell failure, insulin sensitivity, and adipose storage in healthy subjects and T2DM patients. Genomics studies provided information of T2DM therapeutics development [11].

**Figure 1:**
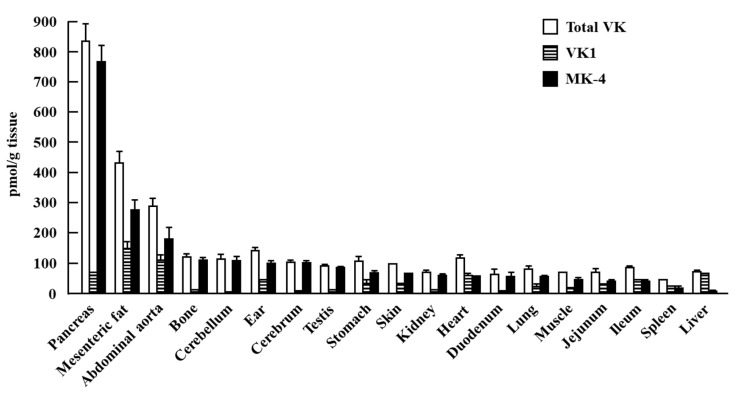
Vitamin K status of male Wistar rat tissues. Rats were fed a standard AIN-93G rodent diet for three weeks. The levels of vitamin K in tissues were determined using fluorescent high-performance liquid chromatography. MK-4: menaquinone-4. (Reproduced from Ref. [17]).

Vitamin K (VK) is a fat-soluble vitamin that exists in two natural forms: VK1 (phylloquinone) and VK2 (menaquinone). VK1 is the major form of dietary VK, which is abundantly present in leafy greens [12]. VK2 is present in dairy products and fermented foods [13], and a homolog of VK2, menaquinone-4 (MK-4), is the major form of VK in animal tissues and is converted from a portion of the ingested VK1 and other menaquinones [14]. The postmortem evaluation of VK status in human tissues, including brain, heart, kidney, liver, lung, and pancreas, revealed that VK1 was stored in all tissues, but with relatively high levels in the liver, heart, and pancreas, whereas VK2 was stored in most of the tissues and had relatively high distribution in the brain, kidneys, and pancreas [15]. In rodents, we previously found that both VK1 and MK-4 are present in all tissues, including the brain, heart, kidney, liver, lung, pancreas, mesenteric fat, abdominal aorta, bone, ear, testis, stomach, skin, intestine, muscle, and spleen (Figure 1). The abovementioned finding was consistent with the results reported from an early study that MK-4 is the major form of VK throughout the body [14] and is observed in high quantities in the liver, bone, brain, pancreas, and reproductive organs, even when animals are fed a low-VK diet [16,17]. The accumulation of VK in the tissues suggested that it has specific physiological roles in the human body.

Several recent studies have mentioned that VK not only plays a role in blood coagulation and bone metabolism but also has specific functions in the regulation of glycemic status; therefore, a greater status of VK may be implicated in mediating a reduced risk of DM. In this mini-review, we outline the current knowledge on the beneficial effects of VK on suppressing the progression of DM.

## 2. Improvement of Insulin Sensitivity and Glycemic Status

Several studies evaluated the effect of VK on insulin response and glycemic status. The evidence indicates that blood VK status is positively correlated with plasma insulin level, and the fasting plasma glucose status was not markedly changed with the intake of VK. In observational studies, at 30 min after glucose loading, the plasma glucose level tended to decrease and the insulinogenic index increased in the high VK intake group, thereby suggesting that VK intake improved the acute insulin response with regard to glucose tolerance [18]. Another study that analyzed the association between the intake of VK1 and insulin sensitivity in older adults showed that higher VK1 ingestion correlated with higher insulin sensitivity and glycemic status in the 2-h oral glucose tolerance test [19], suggesting VK1 intake may have a beneficial effect on glucose homeostasis in adult men and women. Alternatively, in the oral glucose tolerance test, men with lower VK1 intake had decreased insulin levels and an increasing glucose level than men with higher VK1 intake [20,21]. A recent study that used a Mendelian randomization approach indicated that high circulating levels of VK1 are related to a lower risk of T2DM [22]. In intervention studies, a long-term clinical trial of VK supplementation on insulin resistance in older nondiabetic men and women showed that, at the attainable doses of VK1 supplementation for 36 months, the progression of insulin resistance improved in older men but not in women [23]. More recent intervention studies also found that VK1 supplementation for four weeks improved glycemic status and insulin sensitivity in premenopausal and prediabetic women [24,25]. On the other hand, one week of VK2 intake significantly reduced the immunoreactive insulin/plasma glucose ratio following oral glucose loading [26]. Another research team also supported the similar result that four weeks of VK2 supplementation increased insulin sensitivity in healthy young men [27]. In animal studies, rats with low VK intake had poor early insulin response and late hyperinsulinemia after intravenous glucose tolerance test [28]. Furthermore, in an arteriosclerotic rat model with DM, combined administration of VK2 (MK-4) and estradiol reduced the levels of aortic calcium (Ca) and phosphorus (P) and decreased the level of serum glucose while increasing the level of serum insulin, which suppressed the progression of arteriosclerosis with DM [29]. Table 1 summarizes the evidence that shows a significant association among VK status, insulin sensitivity, and glycemic levels.

## 3. Possible Effect of VK Supplementation on Insulin Secretion and Glycemic Status

Based on the evidence above indicating that VK affected insulin response and improved glucose tolerance in clinical and animal studies, the possible mechanisms for the regulation of glycemic status are summarized as follows.

### 3.1. Insulinotropic Effect

Recent studies have highlighted the possibility that two types of agents—incretin mimetics and incretin effect amplifiers—can lower blood glucose via the incretin system. The clinical agents include glucagon-like peptide-1 agonists and glucagon-like peptide-1 analog inhibitors [8,9,30]. Incretin mimetics increase the plasma incretin concentration, thereby contributing to a reduction in the glycated hemoglobin level, fasting blood glucose level, and body weight [31]. In our recent study, we found that MK-4 amplified glucose-stimulated insulin secretion in isolated mouse islets and INS-1 rat insulinoma cells. The findings indicated that MK-4 might function as an incretin-like nutrient via elevation of cAMP levels and resultant Epac2 regulation by using INS-1 cells [32].

### 3.2. Modulation of VK-Dependent Proteins

VK works as a cofactor for microsomal γ-glutamyl carboxylase and has a distinct role in the posttranslational carboxylation of glutamate to γ-carboxyglutamate (Gla) residues of VK-dependent proteins (VKDPs), such as matrix Gla protein (MGP) and osteocalcin (OC), which are involved in the inhibition of vascular calcification and bone mineralization, respectively [33]. Furthermore, these proteins play several beneficial roles in the biological processes and regulate physiological functions. Several studies have found correlations between disease progression and VKDP status, suggesting that VKDPs may potentially emerge as biomarkers for various diseases and, moreover, the VK status may play a crucial role in diseases such as DM [34].

Active MGP is recognized as an inhibitor of vascular calcification, both in vitro and in vivo, and considered to be a biomarker for VK deficiency [35,36,37,38]. Inactive MGP is identified in its carboxylated or phosphorylated forms, including the uncarboxylated MGP (ucMGP), carboxylated but not phosphorylated MGP (dpcMGP), phosphorylated but uncarboxylated (pucMGP), and the fully inactive uncarboxylated, dephosphorylated MGP (dpucMGP) [39,40]. Several studies have described that medial calcification is observed in DM patients, which correlates with the presence of VKDPs [41,42,43,44,45,46,47]. An early report indicated that media calcification was higher in DM patients than in the non-diabetic population [42]. Other studies found that the accumulation of advanced glycation end products correlated with coronary artery calcification in patients with type 1 DM (T1DM) [43] and in those with severe aortic valve stenosis [40]. Furthermore, high ucMGP levels were detected in DM patients and indicated a risk of arterial calcification, which has been observed in non-diabetic subjects [44,47] and T2DM patients [44,45,46].

Another VKDP, osteoblast-specific secreted OC, has been reported to be involved in the regulation of glucose metabolism. Several studies reported a bone-pancreas endocrine loop where insulin signaling stimulates osteoblasts differentiation and OC production, which in turn regulates insulin secretion in pancreatic islet cells [48,49,50,51]. Studies revealed that carboxylated osteocalcin (cOC) modulates hydroxyapatite crystals growth, whereas the undercarboxylated osteocalcin (ucOC) acts as an endocrine hormone in glucose metabolism, energy metabolism and fertility [52,53,54,55,56]. Findings from animal studies suggested that ucOC form improves insulin sensitivity and enhances β-cell functions through the stimulation of cyclin D1 and insulin expression in β cells and adiponectin expression in adipocytes, which ameliorated glucose intolerance in mice [57,58]. However, in clinical trial, participants who received VK1 supplementation had lower serum ucOC levels than the control group, suggesting that the protective effect of VK on the progression of insulin resistance may be mediated by decreasing the ucOC levels, which does not support the findings of the animal studies [23,27]. However, the difference could be species difference between rodent and humans. It is plausible that VK may improve insulin sensitivity and regulate glucose metabolism through modulation of OC and suppression of inflammation [23,26,59,60,61]. Recent clinical studies also supported that OC plays an important role in glucose metabolism by increasing insulin secretion and adiponectin expression [21,24,25]. In addition, Varsha et al. pointed out that VK1 administration may prevent hyperglycemia by protecting pancreatic islets in a streptozotocin (STZ)-induced T1DM model in the rat [62]. A report proposed that decreased level of blood cOC may be a selective early symptom of insulin resistance in obesity, whereas the decreased level of cOC seems to be associated with the appearance of early markers for inflammation accompanying obesity [63]. The association between DM and VKDPs suggests the existence of a novel therapeutic approach for glycemic control.

### 3.3. Prevention of Inflammation

Previous reports have suggested that the suppression of inflammatory cytokines, including tumor necrosis factor (TNF)-α, interleukin (IL)-1, and IL-6 in adipose tissue is associated with insulin sensitivity [64,65,66]. Obesity causes a low-grade inflammation that contributes to the development of insulin resistance and T2DM, suggesting the increased proinflammatory cytokines as key mediators of innate inflammatory responses which contribute to the development of insulin resistance [67,68,69]. Several chronic diseases caused by inflammatory disorders have been associated with VK deficiency [70,71,72]. The evidence shows that VK may attenuate the insulin response and glycemic status through the inhibition of inflammation. In a study that assessed the status of fat-soluble vitamins in patients with chronic pancreatitis, the results indicate that the serum concentrations of fat-soluble vitamins and bone mineral density were decreased in those patients [72]. Similarly, other studies have reported that VK suppressed IL-6 production in lipopolysaccharide-induced inflammation models [59,60]. Moreover, high plasma VK1 concentration and VK1 intake were associated with decreased concentrations of inflammatory markers TNF-α and IL-6 [61]. All of the abovementioned evidence suggests a potential role for VK in the mediation of inflammation and insulin sensitivity.

## 4. Beneficial Effects of VK on Diabetes-Related Complications

The populations with DM that accompany metabolic complications continue to increase worldwide. Diabetes-related complications are generally described as microvascular and macrovascular complications, including retinopathy, kidney disease, neuropathy, and cardiovascular disease (CVD) [73,74]. In this section, we discuss the protective effects of VK on diabetes-related complications.

### 4.1. Cataractogenesis

A recent study reported that the VKDP, active MGP, exhibits anti-calcification and anti-stiffness properties, thereby maintaining retinal microcirculation, which might be considered to be a marker of retinal health [75]. The cataract in rats with STZ-induced diabetes was accompanied by hyperglycemia, high lens aldose reductase 2 (ALR2) activity, accumulation of sorbitol, and the formation of advanced glycation end products within the eye lens that led to diabetes-related cataractogenesis. However, in the rats that were treated with VK1, there was a decrease in the blood glucose level, ALR2 activity, and accumulation of lens sorbitol. The study indicated that VK1 is a potent inhibitor of ALR2 through the inhibition of its substrate-binding site, which suggests a possible mechanism of action of VK1 on diabetes-related cataract formation [76,77].

### 4.2. Diabetic Nephropathy

Increasingly, there is evidence showing that diabetic nephropathy is a serious complication of T1DM and T2DM. Several studies have demonstrated poor VK status and, subsequently, low serum VKDPs levels in patients with chronic kidney disease (CKD). With regard to the VK status, the level of the VKDP, MGP, was highly correlated with the CKD stage [78,79,80,81,82]. There was a strong inverse correlation between the circulating dpucMGP levels and CKD stages, suggesting that MGP as a predictor of mortality in patients with diabetic nephropathy [82,83]; furthermore, the plasma dpucMGP level correlated with albuminuria and proteinuria and was inversely associated with the estimated glomerular filtration rate (eGFR) [84,85]. CKD patients who were undergoing maintenance hemodialysis showed a higher plasma dpucMGP level [86,87]. Moreover, cohort studies revealed that the plasma dpucMGP levels increased with the progression of CKD, especially in patients with CKD Stages 3–5 [88,89]. In addition, other studies have evaluated the risk of increasing dpucMGP levels on renal function. An elevation of the renal resistive index (RRI) that is widely used to evaluate renal dysfunction is associated with adverse renal and cardiovascular outcomes [90]. A recent study indicated that dpucMGP levels correlated with RRI, cardiovascular risk factors, and renal function [91]. The recent Nephrotic Syndrome Study Network cohort study reported that renal MGP expression increased in 5/6 nephrectomy rats [92]. In the same study, the correlation with the MGP levels was investigated by using the information from the kidney biopsies and indicated that eGFR was inversely associated with tubulointerstitial and glomerular MGP mRNA expression in patients with nephrotic syndrome. The tubulointerstitial MGP mRNA expression was strongly correlated with renal inflammation, fibrosis, and acute tubular injury, independently of the eGFR. High MGP mRNA expression was associated with an increased risk for the composite of 40% decline in eGFR and end-stage renal disease [92]. This evidence explains the renoprotective role of MGP and further indicate that VK exerts a beneficial effect on renal function.

### 4.3. Diabetic Peripheral Neuropathy

Diabetic peripheral neuropathy is another frequent and severe metabolic complication of DM; poor glycemic control and dyslipidemia are known to be risk factors for diabetic neuropathy [93,94]. The evidence supports that VK status might be related to nervous system homeostasis. An early study indicated the survival-promoting role of VK on the maintenance of the survival ability of CNS neurons [95]. A report has shown that MGP is expressed by neurons and glial cells [96]. Moreover, the early differentiation and growth of neurons, dendrite formation, development of mature Schwann cells, and myelination are regulated through the interactions of the extracellular matrix and MGP [97,98,99]. Furthermore, dpucMGP plasma levels increased in patients with diabetic peripheral neuropathy and poor VK status, suggesting that MGP plays a role in the homeostasis of the nervous system [46]. Retinopathy and nephropathy are comorbidities that exist with diabetic neuropathy, and, as other reports have indicated, VK exerts a renoprotective effect, which may extend to the prevention of other diabetes-related complications.

### 4.4. Cardiovascular Disease

One of the commonest complications in patients with DM is CVD, including heart failure, vascular disease, and stroke [100]. As mentioned above, in an arteriosclerotic rat model with DM, MK-4 administration with estradiol reduced the levels of aortic Ca and P and suppressed the progression of arteriosclerosis with DM [29]. Moreover, poor VK status has been associated with an increasing risk of CVD in patients with DM [45].

Vascular calcification has long been considered to be a cause of cardiovascular morbidity and mortality. VK plays a role in the modulation of VKDPs that are involved in vascular cell migration, angiogenesis, and calcification [34]. Because a VK deficiency results in increased levels of ucMGP, several studies have implicated ucMGP as a risk factor for vascular calcification and CVD [38,44,45,46,101,102]. A cohort study found that the plasma level of ucMGP was associated with eGFR in patients with CVD [47].

A proof-of-concept study assigned patients with aortic valve calcification and normal renal function to either the VK1 or placebo groups for 12 months. The results indicate that VK administration decreased the serum dpucMGP levels and slowed the progression of cardiac valve calcification [103]. A multicenter family-based cross-sectional study in Switzerland showed that high plasma levels of dpucMGP were independently and positively associated with RRI, after adjustment for several common CVD risk factors [104]. As mentioned above, there are an increasing number of reports indicating that greater VK intake is associated with improved CVD risk factors [105,106], and VKDP activity is associated with CVD through the inhibition of vascular calcification [107,108,109].

### 4.5. Osteopenia and Osteoporosis

DM is a risk factor for osteoporotic fractures, and there is evidence of the increased incidence of osteoporosis in patients with DM. VK plays an important role in the prevention of fractures and the maintenance of bone mineral density and bone quality [110,111,112,113]. In the STZ-induced T1DM rats, the correlation between hyperglycemia and a decrease in femoral weight was confirmed. However, the oral administration of MK-4 for five days a week for 12 weeks in rats prevented the development of hyperglycemia as well as a decrease in the femoral weight, suggesting that VK has beneficial effects on cancellous bone mass in rats with STZ-induced T1DM [114]. The results of randomized controlled trials that investigated the association between osteoporosis and VK in postmenopausal women suggest that MK-4 treatment effectively prevents the occurrence of osteoporotic fractures and a decrease in the serum ucOC level. However, the effect of MK-4 might have occurred with or without an accompanying increase in the bone mineral density [115,116,117,118,119].

## 5. Conclusions

**Figure 2:**
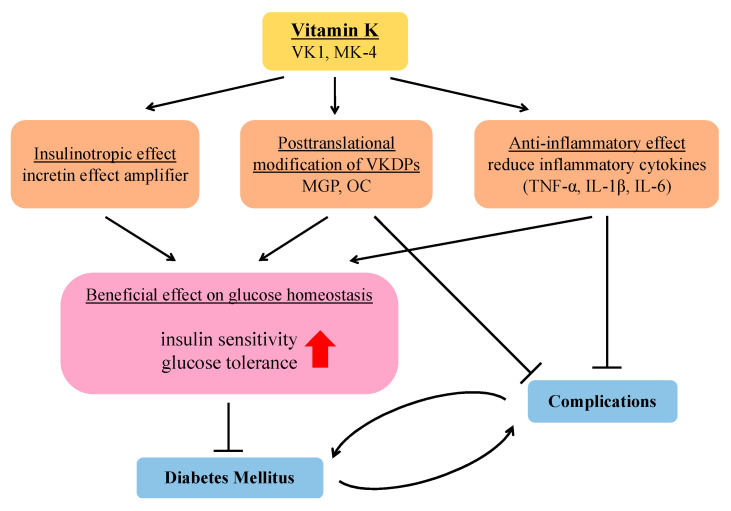
Schematic illustration of the plausible mechanisms of VK on insulin response and glycemic status. MGP: matrix Gla protein; OC: osteocalcin; TNF-α: tumor necrosis factor α, IL: interleukin.

The potential effects of VK on DM and its complications have been demonstrated previously (Figure 2). Reports have mentioned the safety and beneficial effect of VK supplementation in humans [120,121]. However, interventional studies are required to clarify the preventive and protective effects of VK on DM and its complications.

## Figures and Tables

**Table 1:** Summarization of findings from studies on the effect of vitamin K on insulin sensitivity and glycemic status.

Subjects (*N*)	VK dose/VK Status	Period	Outcome	Ref.
**A. Human Studies**				
(a) Observational Studies				
Healthy young men (16)	Usual dietary intake	Acute insulin response A 1-week food-frequency questionnaire to ascertain the daily VK intake	The participants with higher dietary VK intake showed a better insulin response and glucose tolerance.	[18]
Framingham offspring cohort study, adult men (1247) and women (1472)	Usual dietary intake	12 months	In a cross-sectional analysis, higher dietary VK intake was associated with reduced insulin resistance in both adult men and women.	[19]
Adult men (9740) and women (28,354)	Usual dietary intake	10.3 years	Dietary intake of both VK1 and VK2 were associated with a reduced risk of T2DM.	[20]
Elderly men (861) and women (1062) with high cardiovascular risk	Usual dietary intake	Median follow-up of 5.5 years	Dietary VK1 at the baseline was significantly lower in participants who developed T2DM during the study. Increased dietary VK1 intake was associated with a reduced risk of incident T2DM.	[21]
European Prospective Investigation into Cancer and Nutrition (EPIC) cohort study, Diabetes Genetics Replication and Meta-analysis (DIAGRAM), and the UK Biobank (9400 case subjects and 12,182 sub-cohort participants)	Usual dietary intake	EPIC cohort: 1997–2007, DIAGRAM cohort: 2007 (included data from 23 studies), UK Biobank: 2006–2010	Higher circulating VK1 may be causally related with lower risk of T2DM, highlighting the importance of sufficient phylloquinone intake in the human diet.	[22]
(b) Intervention Studies				
Elderly nondiabetic men (124) and women (165)	With or without 500 μg/day VK1 supplementation	36 months	Dietary VK1 supplementation had a protective effect on the progression of insulin resistance in older men.	[23]
Prediabetic women (82)	With or without 1000 μg/day VK1 supplementation	4 weeks	Dietary VK1 supplementation had beneficial effects on glycemic status and insulin sensitivity in premenopausal and prediabetic women.	[24,25]
Healthy young men (12)	90 mg/day menaquinone-4 (MK-4) supplementation	1 week	Short-term VK2 supplementation improved the insulin response after an oral glucose challenge in young men.	[26]
Healthy young men (42)	With or without 90 mg/day MK-4 supplementation	4 weeks	Dietary VK2 supplementation improved insulin sensitivity in young men.	[27]
**B. Animal Studies**				
Rats (unknown)	Low-VK diet (<20% of the required VK1)	Unknown	Rats fed a low-VK diet had poor early insulin response and subsequently increased insulin secretion after a glucose load.	[28]
Arteriosclerotic rat model with DM (unknown)	100 mg/day per kilogram body weight VK2	3 or 6 weeks	VK2 supplementation had a protective effect on arteriosclerosis, by decreasing the aortic Ca and P and the elastin fraction. Rats fed a VK2-rich diet had decreased serum glucose levels and increased serum insulin levels.	[29]
